# Mr. Abernethy, on Aneurysms

**Published:** 1802-02

**Authors:** John Abernethy

**Affiliations:** Bedford Row


### Medical and Phyfical Journal.

VOL. VII.] FEBRUARY, 1802. [NO. XXXVI.

### To Dr. BATTY.

DEAR SIR,

IF the publication of the enclofed Cafe accords with the defign of your work, you will oblige me by inferting it in your next Number. I am defirous that medical men fhould, as foon as poffible, be put in poffeffion of the facts relating to this Cafe, in order that they may judge of them for themfelves, and act in future as that judgment may direct. I am, &c.

*Bedford Row, Jan.* 6. JOHN ABERNETHY.

——— Wrungel, a German, by trade a fugar-baker, of a fickly afpect and flender make, about 5 feet 7 inches high, and near 40 years of age, was admitted into St. Bartholomew's Hofpital, on account of an aneuryfm in the femoral artery, clofe to Poupart's ligament. This had arifen from a ftrain about three weeks before. The tumour at the time of admiffion was of the fize of a fmall orange, and the blood contained in it was fluid; for it could be entirely expreffed from the aneuryfmal fac. At a confultation on the treatment of the cafe, I faid that I did not think a furgeon warranted in tying the external iliac artery, till he was in fome meafure compelled to it by the progrefs of the difeafe, for the following reafons. 1ft. An aneuryfm, in proportion to its increafe and duration, obftructs the paffage of the blood through the natural and principal channels, and obliges it to circulate by other courfes, which are enlarged according to the exigency of the cafe. It feems highly probable, that in proportion to the fize of the artery which is tied, and the magnitude of the part to be nourifhed after that operation, fo will be the degree of previous enlargement to thefe collateral channels, which is neceffary to enfure its fuccefs. On this account the operation fhould be delayed longer in an inguinal aneuryfm than in any other.

2dly, The operation of tying the external iliac artery muft, in the prefent ftate of our knowledge, be confidered as very ferious in its nature, and uncertain in its event. I once tied this veffel veſſel when a man would otherwiſe have bled to death from the femoral artery; and though the limb was nouriſhed, the artery ulcerated. The operation was done a ſecond time in London, and the limb mortified: but no fair practical inference can, I am told, be drawn from the latter caſe, as the operation was poſtponed till mortification was as it were impending.

3dly. There is ſome chance in Aneuryſms of a cure ſpontaneouſly occurring from the cloſure of the artery above by the coagulation of the blood. To cite thoſe inſtances only which have come within my own knowledge, and which it ſeems right to mention, as it increaſes the ſtock of facts before the public; I have known ſuch a ſpontaneous cure take place twice in the poplitæal artery, once in the arteria profunda femoris, and once in the axillary artery. For theſe reaſons it was agreed to poſtpone the operation in the caſe of the preſent patient till circumſtances ſhould appear to demand its performance.

Our poor patient therefore lay in the hoſpital during two months, in which time his diſeaſe gradually increaſed, and his health declined. Towards the latter part of the time he ſuffered a great deal of pain in the front of his thigh, which deprived him of reſt, and the whole limb was largely œdematous. Theſe ſymptoms would naturally ariſe from the preſſure which the aneuryſm muſt make on the anterior nerves and abſorbents of the thigh. The tumour had advanced towards the ſurface, and the ſkin had become ſlightly inflamed, yet the protruding part of the tumour was not of greater extent than when he was firſt admitted into the hoſpital, and no judgment could be formed of that part which was more deeply ſituated, on account of the general ſwelling of the thigh. The blood could even now be expreſſed from the prominent part of the tumonr, and I felt anxious, leſt the obſtruction to the circulation in the main artrey ſhould not have been ſufficient to have obliged the blood to circulate by other channels. It deſerves to be remarked, that the aneuryſm may extend conſiderably beneath the faſcia of the thigh, cauſing pain and œdema by its preſſure, and yet that part which advances towards the ſurface may be of no great magnitude.

The patient's ſufferings increaſed conſiderably during the week preceding the operation, ſo that he declared his preſent ſtate was almoſt inſupportable, and ſolicited that ſomething might be done to change it either for the better or the worſe. He never, however, was able to explain the cauſe of this uncommon degree of anxiety and inquietude.

The operation was undertaken on Saturday the 24th of October. An inciſion of three inches in length was made through the integuments of the abdomen, beginning a little above Poupart's ligament, and being continued upwards; it was more than half an inch on the outſide of the upper part of the abdominal ring, to avoid the epigaſtric artery. The aponeuroſis of the external oblique muſcle being thus expoſed, was next divided in the direction of the external wound. The lower part of the internal oblique muſcle was thus uncovered, and the finger being introduced below the inferior margin of it and of the tranſverſalis muſcle, they were divided by the crooked biſtoury for about one inch and a half. I now introduced my finger beneath the bag of the peritonæum, and carried it upwards by the ſide of the pſoas muſcle, ſo as to touch the artery about two inches above Poupart's ligament. I took care to diſturb the peritonæum as little as poſſible, detaching it to no greater extent than would ſerve to admit my two fingers to touch the veſſel. The pulſations of the artery made it clearly diſtinguiſhable from the contiguous parts, but I could not get my finger round it with the facility which I expected. This was the only circumſtance which cauſed any delay in the performance of the operation. After ineffectual trials to paſs my finger beneath the artery, I was obliged to make a ſlight inciſion on either ſide of it, in the ſame manner as is neceſſary when it is taken up in the thigh, where the faſcia which binds it down in its ſituation is ſtrong. After this I found no difficulty in paſſing my forefinger beneath the artery, which I drew gently down, ſo as to ſee it behind the bag of the peritonæum. By means of an eyed probe two ligatures were conveyed round the veſſel; one of theſe was carried upwards as far as the artery had been detached, and the other downwards; they were firmly tied, and the veſſel was divided in the interſpace between them. Nothing further remained than to cloſe the external wound, which was done by one ſuture, and ſome ſtrips of ſticking plaiſter. The threads of the upper ligature were left out of the wound above the ſuture which cloſed its edges, and thoſe of the lower beneath.

A few remarks on this operation may be permitted. To divide the parietes of the abdomen, puſh aſide the peritonæum, and tie the external iliac artery by the ſide of the pſoas muſcle, is an operation more formidable in ſound, and on its firſt propoſition, than it is in reality. It is performed almoſt without ſhedding blood, ſo that the principal circumſtances of it are very evident. When I formerly performed this operation, I was urged to it by immediate neceſſity: I tied the artery much higher than in the preſent caſe, diſturbed the peritonæum in a greater degree, and, contrary to my own principles, I did not divide the artery. In the preſent caſe, having time to deliberate upon the

 ſteps

ſteps of the operation, I detached merely ſo much of the peritonæum, as enabled me to reach the artery as far as I conveniently could above Poupart's ligament; but not ſo far as to make it difficult to aſcertain that I ſurrounded the artery only with my finger, without injuring any of the adjacent parts, nor ſo far but that I could draw down and diſtinguiſh the artery which I included in the ligature. The remembrance of the ſwelling in the external iliac glands, and of the ulceration of the artery in the former caſe, led to this difference of conduct.

The poor man was greatly exhauſted by the operation, and his leg, which had been chilled by expoſure during the operation, continued very cold for a long time afterwards. It was wrapped up in flannels, to prevent the diſſipation of its own heat; but I would not apply any artificial warmth to reſtore its temperature, leſt it ſhould act as a ſtimulant.

He could not compoſe himſelf after the operation, nor did he ſleep during the night, ſo that on the following day his ſtate was very unpromiſing. His pulſe beat 160 in a minute, his tongue was covered by a dark brown fur; he looked agitated, and a purging took place, which was not reſtrained till the following night by a cordial and opiate mixture. Reſpecting his pulſe, it is proper to mention that it beat 120 moſt days in the week preceding the operation.

His thigh was as warm as that of the ſound ſide, his leg cooler than the oppoſite one, and his foot many degrees colder. He had however perfect ſenſation in his toes, and power of moving them. The leg and foot were rubbed with oil three or four times a day, in order to prevent any ſtagnation in the veins, and to diminiſh perſpiration. It was well covered as before by flannels.

On Monday, the 2d day, (Oct. 26) the pulſe was leſs frequent: he had ſlept a good deal during the night, and ſeemed ſtupified by opium; but on the whole ſo little better, that I concluded he would gradually ſink in conſequence of the ſhock of the operation. The temperature of the limb was a little increaſed. The man however took bread and milk and other food in moderate quantities, whenever it was offered to him: the purging having ceaſed, the quantity of the opiate was diminiſhed. He rather improved in the evening, and reſted well during the night; ſo that on (October 27) the third day after that of the operation every circumſtance wore a favourable aſpect. His pulſe did not exceed 100, and was moderately firm and full; his appetite had increaſed: the temperature of the limb was a good deal augmented, ſo that his foot was ſcarcely colder than that of the ſound ſide; and the œdema of the limb was conſiderably diminiſhed. I now dreſſed his wound, in which he had had not complained of pain, nor of any tendernefs, when the furrounding parts were compreffed. The incifion appeared but as a line, except at the neighbourhood of the ligatures, where it was a little open, and from whence there iffued a moderate quantity of as healthy pus as I had ever feen. The furrounding parts were perfectly natural both in appearance and fenfation. On the 4th day (October 28) he was ftill better: his pulfe 90; his appetite good; his fleep found; and his limb leffening in fize, and increafing in warmth. The ftudents at the hofpital had dreffed the wound before my arrival, and reported that the difcharge was tinged with blood.

On the 5th day (October 29) he was ftill better, his pulfe being but 80 when I counted it. The wound and contiguous parts looked remarkably well, but a bloody fanies was difcharged, which I felt unable to account for.

On the 6th day (October 30) the ftate of his health and limb continued as well, if not improving. The bloody difcharge however had increafed in quantity, infomuch that it ran through the coverings of the wound, and foiled the bed; it had alfo become fœtid. From the firft occurrence of this bloody difcharge I felt confiderable uneafinefs refpecting it. I could not believe that an healthy wound would fecrete fuch a fanies, and I felt apprehenfive left the wound fhould fpread from difeafe. Nothing however took place to confirm this idea. It feemed probable alfo that if the aneuryfmal fac were not entire, fome of the blood being expofed to the air might tinge the difcharge from the wound, and grow putrid. I frequently preffed on the tumour, but could prefs no blood from the wound. In this ftate of uncertainty it was, however, pleafing to obferve, that the patient's health continued in every refpect better than could reafonably have been expected.

The circumftances of the cafe remained very much the fame during the 7th and 8th days after the operation. On the morning of the 9th, (Nov. 2,) when I came to the hofpital, I met Mr. Blicke, who told me that the poor German was dying; intelligence which equally furprized and fhocked me.

He was indeed in a dreadful ftate, appearing like a man far advanced in typhus fever. His pulfe was 150; his tongue covered with a brown fur; his intellect wavering, and the action of his mufcles tremulous. On examining the wound, with a view to difcover the caufe of this great and fudden alteration, and preffing on the tumour beneath Poupart's ligament, I forced out a great quantity of blood, rendered fluid and highly fœtid by putrefaction, infomuch that it inftantly blackened the probe with which it accidentally came in contact.

The caufe and circumftances of the bloody difcharge were now now made clear; the furface of the expofed coagulated blood of the aneuryfm had at firft tinted the difcharge from the wound, then had, by gradual diffolution, been more plentifully commixed with it, and given it a degree of putridity. Till however the whole mafs had become putrid, and had been converted in confequence into fluid, it could not be forced out from beneath Poupart's ligament, when preffure was made on the tumour; nor did it till that period excite inflammation in the furrounding parts by its acrimony, or derange the conftitution by its abforption.

After entirely expreffing the putrid blood, I wafhed out the cyft with warm water, till it returned untinged. The relief which was by thefe means afforded to the poor man, was very ftriking and confiderable. His pulfe became moderate, his intellect clear; he had fome refrefhing fleep, and again took food in moderate quantities. On the following day, when the integuments beneath Poupart's ligament were compreffed, a confiderable quantity of fœtid difcharge and air were forced out. It was not however at all tinged with blood, and appeared to me to be merely the fecretion from the cyft, which had contained the blood. I directed that this difcharge fhould be preffed out, the cavity fyringed, and a poultice applied three times a day; but finding it ftill fecreted in confiderable quantity, I thought it right to make an opening into the cyft beneath Poupart's ligament, to afford it a more ready exit. No abatement in the quantity, or alteration in the quality, was however remarked; it feemed to be fuch as a floughing fore commonly furnifhes.

This fever came on (November 1) on the evening of the eighth day after that of the operation; and I am convinced it would have fpeedily deftroyed him, had not the caufe been detected and removed. The powers of his conftitution rallied again; his pulfe was firm, and often not more than 100; he took fufficient food, and flept moderately well. But the part, as has been faid, did not go on well, and feemed to prevent any increafe of ftrength. For a week I was not without hopes that fome favorable change might happen, but afterwards I loft all expectations, as his already much reduced powers were ftill further declining; neverthelefs, he held out more than another week, when he died on November 16, the 23d day after the operation. A few days before his death both ligatures came away with the dreffings.

### Dissection.

A very flight adhefion had taken place between the figmoid flexure of the colon, and that part of the peritonæum which was oppofite to the wound, but there was no other appearance of that membrane, or of the bowels, having fuffered any inflammation in confequence of the operation. The peritonæum was feparated from the loins, and from the pofterior half of the left fide of the diaphragm, by a confiderable collection of blood, which extended below to Poupart's ligament, and communicated under that ligament by a fmall aperture with the aneuryfmal fac. This opening was fituated in the direction of that crevice which is found between the internal iliac and pfoas mufcles. The only rational explanation that can be given of the formation of this collection is, that the blood had burft its way from the aneuryfmal fac in the vacancy between the mufcles juft mentioned; after which it would readily and extenfively feparate the peritonæum in the manner defcribed. I am inclined to attribute to this circumftance the undefinable difturbance of health, which the poor patient fuffered during the week preceding the operation. It may, perhaps, excite furprize that this collection did not become putrid.

No particular account can be given of the aneuryfmal fac beneath Poupart's ligament, fince it, and the contiguous parts, had floughed in confequence of the irritation of the putrid blood. A fmall aperture had been made by this floughing in the front of the orbicular ligament of the hip joint, and a fmall extent of the thigh bone was, by the fame caufe, deprived of its periofteum.

A bougie was paffed from the lower end of the femoral artery into the fac.

The extremities of the external iliac artery, which had been divided in the operation, were united together by a fine new-formed fubftance; the fides of each extremity were perfectly clofed, and a fmall plug of coagulated blood was found in each.

Having thus given as brief an account as I am able of the circumftances of this cafe, as they appeared to me, I cannot conclude without mentioning the obfervations of others, particularly as they may affift in fuggefting rules of conduct for future operations on fimilar cafes. It has been faid that the irritation of the aneuryfmal bag was probably a fpontaneous occurrence, and not the effect of the acrimony of the putrid blood. But the fuddennefs of this attack, the manifeft exiftence of a caufe fufficient to produce it, and the total abfence of fuch an occurrence in all other cafes of aneuryfm, render this fuppofition highly improbable.

It has alfo been imagined that part of the difcharged blood might have returned from the lower end of the artery. This latter opinion is very improbable, fince after the complete removal of the blood, none returned by that channel; and in the former cafe case which I mentioned, none returned by the inferior part of the artery; though the area of it was ſtill of its natural dimenſions, and unobſtructed. This latter obſervation had tended to diminiſh my confidence in the powers of the communicating channels, and made me wiſh to defer the performance of the operation as long as poſſible. It ſeems evident that in the preſent inſtance it was too long delayed.

It would be deſirable in future to perform the operation before an extenſive diffuſion of blood had taken place; indeed, could the adequateneſs of the collateral arteries for the ſupply of the limb be eſtabliſhed, it would be proper to operate before the artery had burſt.

It deſerves to be conſidered, whether it might not be right at the time of the operation to open the aneuryſmal bag, and remove the blood. I ſhould, however, be inclined to poſtpone this; for, perhaps, no neceſſity might exiſt, no putrefaction might take place. A few days will determine the degree of life of the limb, and will make a wound leſs likely to ulcerate or ſlough. Should ſigns of the putrefaction of the blood enſue, or the probability of ſuch an occurrence become evident, I ſhould think it neceſſary to make a ſmall opening into the aneuryſmal bag for the removal of the contained blood. This being done, if no blood came from the lower orifice of the artery, there would be no neceſſity for tying it. Such are the obſervations that have occurred to me on this ſubject; I have laid both thoſe and the facts before the public as early as poſſible, that medical men might judge of the circumſtances of the caſe themſelves, and form their opinions accordingly.

